# In Search of Risk Factors: The Origin and Early Stages of Cardiovascular Epidemiology

**DOI:** 10.3390/jcdd11010020

**Published:** 2024-01-12

**Authors:** Alessandro Menotti, Paolo Emilio Puddu

**Affiliations:** Association for Cardiac Research, 00182 Rome, Italy; amenotti2@gmail.com

**Keywords:** cardiovascular epidemiology, origins, early stages, risk factors

## Abstract

Based mainly on their personal experience, the authors try to describe the origin of cardiovascular disease (CVD) epidemiology and the problems and difficulties practitioners attempted to tackle and solve during the first few decades of this discipline, which started around the middle of the last century. Beyond identifying the characteristics of those who became CVD epidemiologists, a description is given of the initial structures of the involved studies, participation rates, risk factors measurements and standardization, clinical measurements and diagnostic criteria, mortality data collection and coding, data loading and analysis, plus a number of problems still unsolved at the beginning of the 2000s. Despite many obstacles, and the initial hostility of the medical–scientific establishment, CVD epidemiology represented a revolution in researching in the bio-medical field. In the end, it also affected clinical research introducing the use of the quantitative approach bound to mathematical–statistical procedures. After decades of hard work and the development of a number of innovative tools, CVD epidemiology received its deserved recognition, eventually being accepted as a reputable and independent scientific discipline. Yet, in several countries, especially those from Southern Europe, an academic recognition of CVD epidemiology is still lacking.

## 1. Preface

This paper is not a systematic review of scientific data, but only a personalized history dealing with the origin and the early stages of the discipline that can be defined as cardiovascular disease (CVD) epidemiology. Therefore, the content has nothing systematic, is not necessarily ordered, and mainly reports a number of remembrances largely lived personally. The various chapters will deal with stories and problems related to the first decades since the start and do not progress past the year 2000.

## 2. Introduction

In the past, the origin and causality of major cardiovascular diseases was basically attributed to aging. However, even before the middle of the last century, there were some sporadic observations, usually defined as “geographical pathology”, made by curious investigators, that anticipated the start of true cardiovascular epidemiology. Cornelis De Langen, at the beginning of 1900, was a Dutch internist teaching medicine in Indonesia, who observed that mean levels of serum cholesterol were lower and angina pectoris quite rare in native Indonesians compared to Dutch people, attributing the phenomenon to the vegetarian diet of the natives [1]. Isidore Snapper, another Dutch internist teaching medicine in China about 30 years later, had the advantage of using an electrocardiograph showing the rarity of myocardial infarction in the local population, whose diet was rich in plant food [2]. During the Second World War, there was a substantial decline in cardiovascular diseases in Sweden (a neutral country) and the phenomenon was attributed by Haqvin Malmros, a Swedish internist, to the dietary restrictions suffered in those years [3]. 

The senior author of this report developed an interest in cardiovascular epidemiology in the early 1960s, observing the rarity of myocardial infarction in the wards of the largest hospital of Tripoli, Libya [4], and finding a FAO report from that time. This describes the local diet as limited in calories, which were of 95% vegetal origin [5].

It was only in the late 1940s that the first population studies trying to explain the determinants of coronary heart diseases (CHD) were started. As reference, we can mention three studies that represented the first and somewhat different approaches of CVD epidemiology in the search for what later were called “risk factors”.

1. The Minnesota Business and Professional Men Study was historically the first epidemiological study of CHD, starting in 1947. Its merits were launching the idea of longitudinal population studies, as well as measuring some variables rarely considered later on, such as basal metabolism, the results of the cold pressor test, body-specific weight with complete immersion in water (to estimate the amount of body fat), and exercise testing [6].
2. The study known as the Framingham Heart Study, started in 1950, became the reference of CVD epidemiology for a great number of contributions, such as the initial identification of classical CHD risk factors, the production of the first multivariate analyses and the first practical tools for the estimation of CHD risk [7]. It has passed into history for producing a change in the way we study chronic non-infectious diseases. Incidentally, an article from 1961 from this study introduced the term and concept of “risk factors” for the first time, although they were initially called “factors of risk” [8].
3. The Seven Countries Study of Cardiovascular Diseases (SCS) [9], started in 1958, is the prototype of studies that have compared different cohorts at the international level, located in different countries and cultures. It has documented the existence of large differences in CHD incidence and mortality across different countries, explaining a large part of those differences with basal levels of serum cholesterol, intake of saturated fat, and in part systolic blood pressure. Moreover, it has become the basis for the identification of the so-called Mediterranean diet [10]. Unfortunately, the study could not enroll women and the cohorts were not necessarily representative of the various countries, although there was contrast among them for several characteristics.

Those times, that is in the mid of last century, methodology did not exist, statistics was little known and of difficult use, computers did not exist, or were expensive, slow and of complex use. During the next 50 years or so, many things have been changing and on the basis of a long personal experience we try to describe here the problems met by the investigators in the early phases of cardiovascular epidemiology that was constructed starting about in the mid of last century. Some difference with the present CVD epidemiology research will be occasionally described. Since the purpose is not a systematic review, only few selected bibliographic references will be quoted as examples. This presentation will be mainly focused on methodology and procedures of the early times, only marginally on accomplishments of CVD epidemiology.

Our personal experience involved at least international epidemiological studies mainly developed within the SCS, but also the WHO European Multifactor Preventive Trial of CHD, the WHO Monica Project, plus the Gubbio Population Study and the RIFLE Project in Italy, being involved in several different aspects of these investigations.

A much more detailed and extensive history of cardiovascular epidemiology is available on the web, produced at the University of Minnesota by Professor Henry Blackburn. It offers all possible aspects of this discipline, including descriptions of great studies, biographies of more than 200 investigators from all over the world, technical chapters, interviews, etc. [11].

## 3. Who Are the CVD Epidemiologists?

It might be of interest to understand why a person ends up becoming an epidemiologist and which orientation he/she should have to start this career. There must surely be a substantial scientific curiosity, also stimulated by sporadic observations. There must be interest in problems of general type, including the hypothesis that difference in the amount of a disease across populations may include hidden information about the possible causes of the disease. Moreover, there should be a belief in the hypothesis that mass diseases should be explained by mass phenomena. Finally, he/she could be affected by the fascination for prediction and the hope for prevention.

Many investigators of the first generation (those starting their activity before the 1980s) were actively working in prototypal studies while the discipline was still under construction from the theoretical point of view. Moreover, in those times it was necessary (for being a good epidemiologist) to acquire or already have at least elementary competence in several disciplines, even those not strictly medical–clinical. It was not only the question of knowing about epidemiology, whose doctrinal knowledge was under construction, but also some elements of clinical science. It was necessary to be competent in the use of some devices such as an electrocardiographic machine or a spirometer. Some knowledge of biochemistry, nutrition science, mathematics, statistics, and computer use were useful together with some demography, sociology, and psychology, and all this was complemented with some interest and curiosity in geography, history, culture, and languages mainly when the work was conducted abroad. 

All this made the activity rather attractive due to its complexity, and the need to consider several disciplines induced people to feel more complete and not like super-specialists. CVD epidemiology was something innovative that, in terms of research, requested the identification of a question to answer, usually bound to the possible causality of disease; it promoted longitudinal studies. It needed pre-condition statistics, sampling, and principles of measurement. It was based on teamwork of multi-disciplinary character, within which all were able to perform almost all tasks, including a relevant amount of field work, coding procedures, and manual checks of data. It imposed, alas, the need to use computers with little power to perform important analyses, usually long and complex, to reach valuable conclusions. All this required great enthusiasm. Indeed, those of the old generation moved from a clinical environment to epidemiological and population-based studies. Moreover, they usually worked in the same scientific environment despite being sometimes competent in different disciplines, a situation that today is definitely rare.

Those of the intermediate generation (those starting activity during the last 20 years of last century) were almost born into epidemiology, finding the discipline already organized and a minimum potential for a career. Their activity was definitely made easier.

Those of the present generation (those starting their activity in the years 2000), or at least some of them, seem to have been conceived as such. In fact, once becoming able to command an informatic technique, they found mountains of data already available and ready to be analyzed. In fact, today many aspects have changed, probably in favorable but also in unfavorable directions. The fact is that there are many epidemiologists who are not physicians, who have never seen a single case of the diseases on which they work and have never made a single measurement of the study variables that they analyze using easy-to-manage statistical packages.

Actually, mainly during the 1960s, the London School of Hygiene and Tropical Medicine became a major education and training center for CVD epidemiology, and many of those of the first generation benefited from its offers including technical, practical, theoretical and mixed courses of short- and long-term duration. Most pupils were medical doctors transitioning toward CVD epidemiology. 

## 4. Evolution of CVD Epidemiology

Attaining formal acknowledgement from the official medicine, academy and public health was not easy, except in the USA and Northern Europe. Still, in the 1980s some “wise clinicians” thought that epidemiologists were going to study some obvious facts in a complex way and reach obvious conclusions of no interest. Some colleagues working in other disciplines thought that epidemiologists followed absolutely useless questions that did not need answering and that they produced everything except research. A young boy, son of a renowned epidemiologist, when questioned by a friend, said that the profession of his father consisted in “counting the deaths”. At least he was not so wrong.

On the other hand, for a long time many clinicians were convinced that hypertension started from a systolic blood pressure of 180 mmHg or more and high blood cholesterol of 300 mg/dL or more. More recently, the same people started “to preach”, saying that a healthy systolic blood pressure should be less than 130 mmHg and serum cholesterol less than 200 mg/dL, forgetting and disregarding that these concepts were demonstrated by CVD epidemiology several decades ago. Again, in relation to the temporal evolution of the discipline, it should be recalled that yesterday the environment of CVD epidemiology was characterized by the existence, in the same institution, of multidisciplinary competences and was driven by somebody who had passed through experiences in various disciplines. Today, the environment seems still multidisciplinary, but in fact it is only minimally so or not at all, this not being necessary anymore. It relies on computers, but it is necessary to exploit external specific competences of specialists (hemato-chemical laboratories, genetic testing, etc.) that are frequently not aware of what they are involved in and do not always understand or share the principles of research that characterize epidemiology. Tomorrow, the investigators will probably use only computers, no competence will be needed, and the collection of data will be entrusted to machines (or avoided), and the task will only involve performing analysis to exploit routine data collected in everyday medical practice. An example of this apparently foolish statement can be found in a paper produced by investigators of the Kaiser Permanente Foundation, Berkeley (a medical insurance organization), on a cohort of people insured there. In this research, the genetic tests were performed by a completely different organization, while all the other traditional risk factors were obtained via questionnaire (!) or via routine examinations performed within insurance plans [12]. 

Despite all this, epidemiology in general and the CVD in particular have started a revolution in the way of thinking in many areas of medical sciences, and some acknowledgements started to arrive at the end of the last century. Eventually, it was recognized that health and disease problems cannot be tackled at only an individual level and after the beginning of a disease, but also at population level and before the occurrence of a disease.

The introduction of new concepts and the demonstration of the predictive and sometimes causal role of characteristics measured before the occurrence of an event—that is, risk factors—has represented a conceptual revolution in the approach to causality previously called “etiology”, which was considered a deterministic process. All this has introduced the concept of probabilistic association that many do not like. However, for the moment there is no different or alternative theory, and its value is confirmed at least for pragmatic purposes since even nuclear physicists have adopted it. It so happened that investigators started to understand that mass diseases occur due to bad cultural habits (and mass habits) and that only systematic changes to those habits may drive to a substantial decrease in the CVD epidemic, although it is probably impossible to hope for an eradication. This research methodology and the use of medical biostatistics developed in the area of chronic disease and CVD epidemiology in particular have invaded all other medical disciplines, serving as the bases for a more scientific and less approximated approach to medical research.

An excess of enthusiasm induced the invention, more recently, of the term “clinical epidemiology”. This represents a contradiction in terms, but explains the influence that epidemiology has had on the study of clinical problems as well. Then, as the next exaggeration, a renowned theoretical epidemiologist stated that epidemiology does not exist anymore since only bio-medical–clinical–health quantitative research exists that, among other things, can deal with population studies. 

## 5. Structure of Studies

In the majority of cases, a classical CVD epidemiology investigation included the identification of cohorts or population groups, an entry examination of all participants with measurement for potential risk factors and baseline prevalence of CVD, subsequent periodical examinations, and a search for new incident CVD events and mortality data. In this case, the statistical units for analysis were made up of single individuals. In rare cases, multiple cohorts were enrolled and examined in the same way, but the statistical unit was made up of the various cohorts producing the so-called “ecological analyses”.

There were also some case-control studies, but their value was minimal compared with longitudinal population studies and they were soon dismissed, being used only for clinical investigations.

## 6. Participation Rate

In the past, there was much attention paid to obtaining a high participation rate at entry examination once the population sample to be studied had been defined. This is explicitly said in monographs of the Framingham Heart Study [7] and the SCS [9] in order to avoid uncontrolled selection of those examined. For example, in the Italian rural areas of the SCS, two employees of the local municipality were enrolled to invite the selected subjects one by one and convince them to participate. Moreover, direct help was obtained from the local lord mayor, his political party, and from the local church priest. The team was working every day of the week including Monday (the free day of barbers), Saturday (the free day of bank employees), Sunday (the free day of most people with employment), and Wednesday (the day of the local market). All these efforts allowed us to obtain participation rates in the order of 98–99% [13]. This does not mean that the study cohorts should not be varied and comprehensive from several points of view, but that once defined the maximum effort should be made to obtain a very high participation rate. 

Presently, it seems that this problem is rarely mentioned or even not considered, with many likely supposing that examining large numbers of subjects may dilute the possible distortions due to low participation rates. It is in fact quite difficult to find publications where attention and reporting of participation rates are presented, although there are some valuable exceptions such as those quoted here [14,15], that reported 90 to 95% participation rates.

## 7. Risk Factor Measurements and Standardization

In the early stages of CVD epidemiology, much attention was given to the principles of measurement and the standardization of methods. These problems are clear when reading the monographs of the Framingham Heart Study [7] and of the SCS [9]. For example, in the SCS (in the middle of last century) it was decided to adopt only measurements that were clearly standardized and validated via strict quality control. Despite the large number of measurements actually adopted, many more were discarded since at that time it was impossible to reach valuable standardization procedures. Potentially interesting variables such as blood glucose, alfa-lipoproteins (the term of those times to identify HDL cholesterol), serum triglycerides, some indicators of coagulation, chest X-ray and others were considered in pilot studies but finally discarded. On the other hand, centralized operations were the serum cholesterol assay, the interpretation of electrocardiograms, the final allocation of diagnoses made in field clinical examinations, and the coding of causes of death.

A few examples of the standardization of common risk factor measurements might be of interest. In most studies, there was a peculiar action for the standardization and quality control of serum cholesterol assay that was usually measured using the Abell–Kendall technique, while standardization relied on contact of and interaction with reference centers such as those of the CDC in Atlanta, USA, and that of the WHO in Prague, Czechoslovakia.

Measurement of blood pressure was also considered, and the teaching and testing of the field operators was common. This was mainly for the digit preference problem and frequently for the need to report measurements of 2 by 2 mmHg. A useful tool for that purpose was the London School of Hygiene cassette, which served for instruction, training and testing. 

A great help to field epidemiologists came in 1968 with the publication of the WHO manual of Cardiovascular Survey Methods [16]. It contained detailed procedures for the measurement of a series of anthropometric characteristics and blood pressure, procedures for recording and reading electrocardiograms and spirometry tests, techniques for measuring serum cholesterol, dietary and motion habits, and others. 

Nowadays, some of these problems seem to have been overcome. For example, many semi-automatic devices for blood pressure measurement can be found on the market and some of them have been tested towards traditional human Hg-sphygmomanometer readings and validated according to strict rules imposed by the European Society for Hypertension [17]. Nowadays, hemato-chemical assays are run by complex machines that are able to perform many different measurements almost at the same time in a rather automatic way. Initially called “auto-analyzers”, they now incorporate procedures that warrant standardization and quality controls without the need for external reference centers. Presently, this seems to be a generalized situation that determines a definite improvement after the initial cumbersome processes needed in the middle of last century.

## 8. Clinical Measurements and Diagnostic Criteria

The complexity of clinical information, measurements, and the final diagnosis in field epidemiology created serious problems for the early CVD epidemiologists. Each research group followed different approaches including the use of questionnaires, physical examination, blood pressure measurement, and possibly an ECG tracing. However, the combination of these components to reach a final diagnosis could not be standardized across studies.

Some help came from the spread of the Rose London School of Hygiene questionnaires on angina pectoris, myocardial infarction, and intermittent claudication [16], as well as those on bronchial symptoms by Fletcher [16]. They had little value in terms of reaching a valuable clinical diagnosis, but they allowed us, if properly administered, to assure a minimum comparability across different individuals and studies. Another step towards homogenization of the findings was the publication, in 1960, of the ECG Minnesota Code, a quantitative system for ECG reading based on measurements of various waves, intervals, etc. [16,18]. This approach needed a lot of training and testing of coders–observers in order to obtain a minimum of homogeneity in the final codes. For decades, a Reference Center for ECG Minnesota Code was active at the University of Minnesota, providing teaching, testing and coding for third parties. Another reference center was established by the WHO in Budapest, Hungary. More recently, a computer program was produced to read the ECG by the Minnesota Code rules showing a good performance comparable with the one provided by expert human coders using the visual approach [19]. This became useful in terms of sparing manpower and time.

In the 1970 monograph of the SCS, the combinations of those questionnaires with ECG findings read by the Minnesota Code allowed us to produce clear criteria for the definition of 6 categories of prevalent CHD and 9 categories of incident CHD events [20]. Detailed but different diagnostic criteria for CHD were created by the Framingham Heart Study [7].

Nowadays, several studies take CVD incidence data directly from routine hospital (national) archives, excluding the need to validate those diagnoses. An example is that of a study in New Zealand, where incidence data were taken from national morbidity–mortality collections [21]. Of course, hospital official diagnoses now should be more valid than those of several decades ago or when those archives were not available, but this seems to be a revolution in epidemiology studies, where routine data are used instead of those collected directly by the investigators as was the case at the beginning of CVD epidemiology. It might be wise to adopt validation studies before fully accepting the new methods. 

## 9. Mortality Data Collection and Coding

Those who started CVD epidemiology had the problem of collecting and coding mortality data that frequently became the only end-point available for analysis. At the beginning, through relatively simple public relations operations, it was possible to obtain from proper sources (Municipal Offices, Statistical Offices, etc.) the list of those who died in a given time period, together with written causes of death (or even WHO-ICD codes). However, the reliability of causes of death at those times was at least doubtful. Several studies took them as adequate, accepting some gross mistakes, typically as follows: if the first cause were cardiac arrest, the second be brain metastases, and the third lung cancer as such, the case would have been classified as a heart disease, possibly a CHD sudden death, and all this would have been wrong. Some studies created clinical committees to review and agree about the final cause of death.

A different approach was taken within the SCS where, beyond the availability of death certificate, a major effort was made [13] to also exploit information from repeated field examinations (including ECG tracings), clinical records from hospital and other sources, interviews with family and hospital doctors and with relatives of the subjects, and any other possible source of information. In this way, the final cause was reached on the basis of a larger amount of information and then coded using the WHO-ICD-8 [22]. This approach probably allowed us to better identify and classify CVD and other causes of death, at least within the limits of epidemiological procedures, anticipating by years the concept and structure of the verbal autopsy instruments produced by the WHO many years later [23]. Finally, a compacted re-classification of causes of death, largely dedicated to CVD, was created in order to distinguish possible different etiologies. Moreover, in the presence of multiple causes of death and uncertainty about the principal one, a rank criterium was adopted with violence, cancer, CHD, stroke, and others in that order [24]. The job was performed by two investigators for the first 14 years and then by only one of them until year 60 of follow-up.

A serious problem in collecting and using mortality data, even for research purposes, derived from the more and more strict rules of “privacy” now spread all over the world. Even in countries well organized for the identification and classification of “research institutions” and “research purposes”, obtaining those data became a nightmare due to the complexity of bureaucratic procedures, a number of limitations, and the final uncertainty of outcome. This was a great damage to conducting follow-ups with mortality data collection and use.

## 10. Data Loading and Analysis

In the early times of CVD epidemiology, the collection of data was a question of paperwork. There was the problem of loading the data in a kind of memory consisting of “punch cards” with the option for 80 or 90 columns. The job was long and tedious and frequently performed by the same investigators engaged in the collection of data. Nowadays, the data collected are immediately entered during the field work and instantaneously memorized on a computer that chooses the proper cells. This also represents a way to spare time and manpower what improves precision.

Again, in the early times, analysis of data was nightmare. In fact, personal computers did not exist, nor did valuable statistical packages, while big computers were rare, expensive, sometime slow, difficult to use and again statistical programs did not exist, with the consequence that a program had to be devised, written, and tested for each specific analysis. The only resource was the use of the sorting machines that could handle the punch cards, but they were noisy, slow, and practically only capable of performing “counts”. 

At the end of the 1960s and beginning of the 1970s, the first simple desk computers became available. The Olivetti 101 produced in Italy is usually considered the prototype of this kind of computers. It could perform different kinds of computations, but the available memory was extremely limited and specific statistical programs had to be written ad hoc. The limited memory imposed the requirement to enter the variables and use them immediately for some operations (say additions, multiplications, etc.) since there was not enough space to retain them. The Wang desk computer produced in the USA was definitely more powerful in view of performing complex analyses. At the University of Minnesota, a Wang computer was connected with an electric typewriter, allowing us to print the results instead of copying them from a small screen. Again, at the University of Minnesota at the beginning of the 1970s, a powerful CDC 6000 could be used to solve the multiple logistic function from the SCS data, requiring only 6 min to produce the results. However, the program was written on an enormous number of punch cards, corresponding to more than 20 kg. The program in that shape was imported to Italy, where it was fed into the most powerful computer available at the National Institute of Public Health (Istituto Superiore di Sanità). It was a great IBM machine made of 5–6 large cabinets, plus an enormous machine used to enter the punch cards and a similarly enormous printer. The machine could not be purchased but only rented, with an annual cost of about US $ 550,000 during those times. Despite all this, the multiple logistic function could be solved in about 50 min! Nowadays, a desk computer (desktop) weighing less than 2 kg, with a cost not exceeding 1500 US $, performs the same computation in a few seconds using a program loaded onto a small pen weighing few grams.

In terms of speed, if the task consists of covering a distance of 5 km, a person moving with a brisk walk travels it in 50 min, while a supersonic jet flying at the speed of 3600 km per hour covers it in 5 s. This is the difference between a large old IBM computer and a modern desk computer (desktop). Moreover, nowadays many easy statistical packages exist, allowing quick performance of analysis.

The search for predictive risk factors of CVD events was initially performed computing rates in arbitrary classes of one risk factor at a time, but this was absolutely unsatisfactory since many risk factors play their role in combination with and/or are related to one each other. The multiple logistic function was the first valuable tool to estimate risk as a function of multiple risk factors and the related Walker–Duncan program became very popular. That was the one mentioned above in relation to the computer power and speed. However, soon after that, it became clear that the availability of many events spread during long time periods could not be evaluated in a proper way by a model that did not consider the role of time. Therefore, the subsequent availability of models including the time to an event allowed us to tackle this problem. The Cox proportional hazards model, the Weibull model, the Poisson model and others became commonly used. They were all developed scientifically and popularized via application, especially in epidemiology, in the 70s, coming along with the rapid availability of computing power due to widespread research in informatics that also produced steeping reductions in costs.

## 11. Preventive Trials

CVD epidemiology started as an observational discipline whose merits were to provide facts consisting in the statistical relationships of risk factors (as possible causes) with CVD events of various types (as possible effects). However, this did not demonstrate a causality relationship that requested a final experiment to prove it, although on some occasions the majority of the causality criteria [25] were met. The next step was the start of preventive trials, where the possible effect of the possible cause was tested by changing the levels of the risk factors in the hope of seeing a real reduction in risk. This new approach needed further complex methodologies and procedures (for example, the randomization of patients into treatment or control groups and the double-blind procedure that prevents both patients and investigators from knowing who the treated and control subjects are) higher costs and new terms of ethical rules. 

Some of these studies were run on large groups of populations and were based on health education and community approaches, mainly targeted toward stopping smoking, changing dietary habits, and starting antihypertensive drug treatment, like the North Karelia Project [26]. The majority were run on groups of single individuals, frequently selected on the basis of their high risk. It is worth recalling some of those that represented the prototypes in this area, such as the Veterans Administration Cooperative Study for the treatment of hypertension [27], the Oslo Study that demonstrated the combined causal role of serum cholesterol and smoking [28], and the Helsinki Heart Study, perhaps the first using drug treatment of dyslipidemia [29]. The long story of the causal role of high serum cholesterol was later reviewed in relation to its controversy [30]. As for the mentioned prototypal observational studies, we do not enter into detailed description of many more recent trials and their results.

## 12. Some Open Problems

Several problems of a different nature were still open at the beginning of the 2000s, being substantially unresolved during the previous century. A few examples are reported here.

**Saturation effect.** In the initial phases with the use of multivariable predictive models, there was great interest in using many risk factors as predictive covariates. However, soon it was shown that the discriminant power of many risk factors was impaired and associated with a flattening of indicators of predictivity when an even relatively small number of risk factors was added in the model [31]. The problem is still present when the issue is tackled with the use of R curves whose improvement, after a while, becomes irrelevant whatever new factors are added.

**Metanalysis.** One of the relatively novel analytical approaches that started to be used several decades ago, and then became of more widespread use, was metanalysis. The procedure consisted in collecting and combining data from many different and already published population studies in order to evaluate the relationships of risk factors with events based on very large numbers. This allowed to perform analyses on millions of subjects and soon dedicated computer programs became available. This procedure was invented in a rough way at the beginning of 1900 but became widely used around the 1980s.

After the initial enthusiasm, serious critics of this type of analysis have emerged. In fact, the material is, by definition, heterogeneous, and combining data collected and coded with largely different methodologies may prevent a serious evaluation of final findings. Another reason for criticism was that the attempted efforts to homogenize data may be impossible or useless. Clearly, the (probably undemonstrated) idea was that using large groups, whatever their origin, may cancel the heterogeneity of the overall composition of the final group analyzed. The fact is that competent statisticians in this field recommend checking the presence of excess of heterogeneity and, in this case, avoiding analysis. Apparently, however, this advice is almost always neglected. 

Another serious problem is that the choice of the end-points to be analyzed (almost only mortality data) does not take into proper account the different durations of follow-up and the possible different “etiologies” of subgroups of CVD since the decision is usually in the hands of investigators who look uncritically to the WHO ICD codes and whose main competence is almost always only in informatics. For example, in a giant metanalysis dealing with serum cholesterol published in 2007, CVD mortality was classified in a probably wrong way. In fact, the ischemic heart disease (CHD) group contained subgroups of syndromes that can hardly be defined as coronary syndromes and the large group of the other circulatory events contained a mix of etiologically and not etiologically defined conditions whose relationships with risk factors are surely different [32]. 

In a similar way, many analyses include all possible CVDs, simply because they belong to the same anatomical–physiological system, disregarding the fact that the etiology of subgroups are likely very different. This would be the same as mixing all diseases from mouth to rectum simply because they belong to the same anatomical–physiological system of the digestive tube.

Moreover, hazard ratios obtained after these giant analyses are frequently not different from those obtained from good studies made using much smaller samples. In any case, the outcome might be driven by the characteristics of the most numerous groups enrolled in the metanalysis. 

**Choice of end-points.** The fact is that, initially, most studies were focused on CHD, while later the interest expanded to other types of CVD. In many cases, this approach consisted in using all kinds of CVD conditions, or at least those supposed of arterio- or atherosclerotic origin. In this way, at least for conditions listed in the ICD as ischemic heart disease, all kind of stroke and “symptomatic heart disease”, mainly involving heart failure (without any evident etiology), were used in pools. Our position was to disentangle at least the true typical CHD from other non-typical heart diseases of uncertain etiology [33] and consider strokes as a third independent group [34]. This was performed simply because those three groups have documented different risk factors (and probably etiologies as illustrated by elements of competition) and different natural histories [35]. 

**New risk factors.** After the first 3 decades of active CVD epidemiology, a relatively large number of major risk factors were well established (say blood pressure, serum cholesterol, smoking habits, diabetes and many more). Still, there was a phase when the search for “new” risk factors became fashionable and some strange characteristics were added to the list, such as indicators of coagulation and thrombotic processes, rare lipoproteins, homocysteine, estrogens deficiency, chlamydia pneumoniae, helicobacter pylori, C-reactive protein, and other indicators of inflammation. 

The initial enthusiasm was rapidly spent when it was shown that many conclusions came from poorly reliable case-control studies, that their independent predictive power was not large enough, and that many of them could be intermediate stages of the disease process but not real causes. The example dealing with homocysteine is probably valid for other so-called new risk factors [36].

**Genetics.** Genetic measurements were not part of the early stages of CVD epidemiology, but recently there has been a large number of contributions. However, in addition to studies that claim great discoveries [37], there are others that cast doubts about the present possibilities of exploiting such findings for practical use, mainly because the addition of genetic markers does not seem able to substantially improve the performance of traditional risk factors [38]. The problem will probably be solved when new markers with no relationship to the traditional risk factors are hopefully identified.

**New predictive models.** The need for new predictive statistical models was always felt to be necessary in view of improving the specificity and sensitivity of risk estimates and allowing the use of larger numbers of risk factors. However, the waiting time was rather long.

An innovative approach to improving predictive analyses relied on neural network procedures. However, it did not attract much attention from CVD epidemiologists and an attempt made by our research group did not show substantial improvements in events prediction when compared with traditional simpler models [39]. More recently, some new approaches seem able to answer these questions, but a final judgement cannot be given at this moment [40]. We are adding to these initiatives the promises of artificial intelligence, whose capabilities have not yet been properly tested in this area.

## 13. Conclusions

This short, mainly personal historical review of the early stages of CVD epidemiology suggests that this relatively new discipline, together with the other parallel ones in primarily the areas of cancer and/or renal epidemiology, represents a revolution in performing research in the bio-medical field, looking to population instead of single individuals, to still-healthy people instead of ill people, and searching for causes in still-healthy subjects before following them up for years. Moreover, it also valuably contaminated clinical research with the quantitative approach bound to mathematical–statistical procedures being used. Initially, this discipline suffered from an absence of theory and practice, which were created together with the development of the first pioneer’s field studies, plus the declared hostility of the medical–scientific establishments. It is a tiny substrate of this hostility that remains in most South European countries, where there is an unfortunate lack of academic independence and limited recognition of CVD epidemiology as an autonomous discipline. It incorporated into but not practiced by cardiologists and internists with devoted interest in hypertension mainly, and this greatly interferes with the pharmaceutical industry. Still, after decades of hard work and with the development of a number of innovative tools, CVD epidemiology has emerged and now fully deserves recognition as an original scientific discipline.

## Data Availability

The original data are not publicly available. However, research projects are evaluated centrally by an ad hoc committee.
